# Rivaroxaban and other novel oral anticoagulants: pharmacokinetics in healthy subjects, specific patient populations and relevance of coagulation monitoring

**DOI:** 10.1186/1477-9560-11-10

**Published:** 2013-06-28

**Authors:** Wolfgang Mueck, Stephan Schwers, Jan Stampfuss

**Affiliations:** 1 Bayer Pharma AG, Clinical Pharmacology, D-42096 Wuppertal, Germany

**Keywords:** Rivaroxaban, Population Pharmacokinetics, Dabigatran, Apixaban, Coagulation Monitoring

## Abstract

Unlike traditional anticoagulants, the more recently developed agents rivaroxaban, dabigatran and apixaban target specific factors in the coagulation cascade to attenuate thrombosis. Rivaroxaban and apixaban directly inhibit Factor Xa, whereas dabigatran directly inhibits thrombin. All three drugs exhibit predictable pharmacokinetic and pharmacodynamic characteristics that allow for fixed oral doses in a variety of settings. The population pharmacokinetics of rivaroxaban, and also dabigatran, have been evaluated in a series of models using patient data from phase II and III clinical studies. These models point towards a consistent pharmacokinetic and pharmacodynamic profile, even when extreme demographic factors are taken into account, meaning that doses rarely need to be adjusted. The exception is in certain patients with renal impairment, for whom pharmacokinetic modelling provided the rationale for reduced doses as part of some regimens. Although not routinely required, the ability to measure plasma concentrations of these agents could be advantageous in emergency situations, such as overdose. Specific pharmacokinetic and pharmacodynamic characteristics must be taken into account when selecting an appropriate assay for monitoring. The anti-Factor Xa chromogenic assays now available are likely to provide the most appropriate means of determining plasma concentrations of rivaroxaban and apixaban, and specific assays for dabigatran are in development.

## Introduction

In recent years, the scope for effective management of venous and arterial thromboembolic diseases has been enhanced by the advent of novel oral anticoagulants (OACs) that, unlike traditional oral vitamin K antagonists (VKAs) [1], are given at fixed doses and have a lower potential for drug and food interactions [2]. These agents show similar or improved efficacy and safety profiles compared with VKAs, such as warfarin, and established parenteral agents, including unfractionated heparin and low molecular weight heparin [2].

The currently licensed novel OACs are rivaroxaban (Xarelto®, Bayer Pharma AG and Janssen Pharmaceuticals, Inc.), dabigatran (Pradaxa®, Boehringer Ingelheim International GmbH) and apixaban (Eliquis®, Bristol-Myers Squibb and Pfizer EEIG). All three of these agents, and others in development, are under investigation for the management of multiple thromboembolic disorders. Rivaroxaban, a direct Factor Xa inhibitor, is now approved in the European Union (EU), United States (US) and elsewhere for the prevention of venous thromboembolism (VTE) in adults who have undergone elective hip or knee replacement surgery at a dose of 10 mg once daily (od) given for 2 weeks (knee) or 5 weeks (hip) [3,4]. Apixaban [5], another direct Factor Xa inhibitor, and dabigatran [6], a direct thrombin inhibitor, are now also approved in the EU for the same orthopaedic indication. In addition, rivaroxaban is approved for the prevention of stroke and systemic embolism in adults with non-valvular atrial fibrillation (AF) (20 mg od; EU and US) [3,4], and for the treatment of deep vein thrombosis (DVT) and pulmonary embolism (PE), and prevention of recurrent DVT and PE in adult patients (15 mg twice daily [bid] for 3 weeks followed by 20 mg od; EU and US) [3,4]. Apixaban and dabigatran are also licensed to reduce the risk of stroke and systemic embolism in patients with non-valvular AF in Europe and the US [5-8]. Rivaroxaban has also recently been approved in the EU for the secondary prevention of acute coronary syndrome (ACS); rivaroxaban administered with acetylsalicylic acid (ASA) alone or with ASA plus clopidogrel or ticlopidine is indicated for the prevention of atherothrombotic events in adult patients with elevated cardiac biomarkers after ACS [3].

The novel OACs have mechanisms of action that attenuate thrombotic processes via direct targeting of specific factors in the coagulation cascade [9]. An essential component of the clinical development of these agents has been a full characterisation of their pharmacokinetic (PK) and pharmacodynamic (PD) profiles. In particular, the phase III clinical trial programme for rivaroxaban has been supported by a comprehensive set of phase I and II studies evaluating PK and PD in both healthy subjects and patients receiving the drug for active prevention or treatment of thrombosis [10-12]. These studies have demonstrated the predictable PK and PD properties of rivaroxaban that allow fixed oral dosing regimens to be followed, as well as characterising other important aspects, such as limited clinically relevant drug–drug interactions [10].

Because of their predictable PK/PD profiles, rivaroxaban and other novel OACs do not require routine coagulation monitoring [2]. However, there are circumstances in which it may be necessary or desirable to measure their anticoagulant effect or the plasma levels of these drugs [9]. In such cases, it is important to appreciate the PK properties of the drugs and their influence on coagulation assays. The objectives of this review are to provide an overview of the mechanism of action of rivaroxaban, to summarise its known PK and PD characteristics in healthy subjects and patient populations, and to give information on potential laboratory tests for rivaroxaban. Dabigatran and apixaban are discussed where relevant differences exist.

## Mechanism of action of novel oral anticoagulants

**Figure 1:**
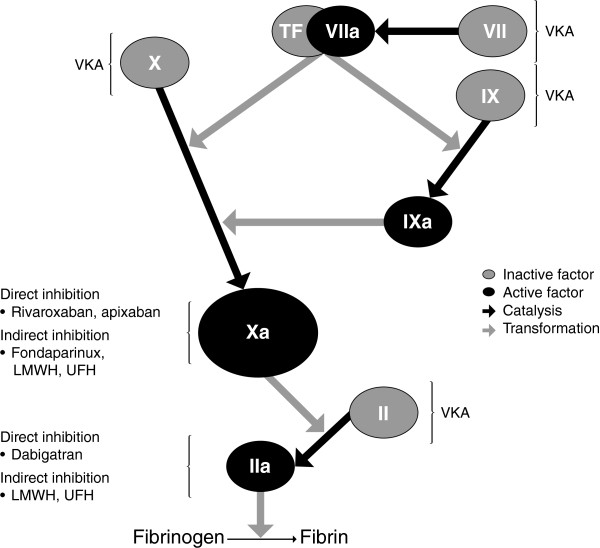
**The coagulation cascade and targets of anticoagulant agents.** LMWH, low molecular weight heparin; TF, tissue factor; UFH, unfractionated heparin; VKA, vitamin K antagonist [9].

Unlike traditional anticoagulant agents, the VKAs and heparins, novel OACs have been designed to inhibit specific single targets in the coagulation cascade (Figure 1) [9]. Rivaroxaban and apixaban directly inhibit Factor Xa, whereas dabigatran targets thrombin (Factor IIa). In addition, the parenteral agent fondaparinux indirectly inhibits Factor Xa (Figure 1).

Rivaroxaban, the first oral, direct Factor Xa inhibitor to be developed, was designed to specifically target Factor Xa for several reasons. Factor Xa occupies the crossroads between the intrinsic and extrinsic pathways in the coagulation process [13] and is responsible for converting prothrombin (Factor II) to thrombin (Factor IIa) [14]. In preclinical studies, rivaroxaban was found to be highly selective for Factor Xa, with an inhibitory effect >10,000 ­fold higher than for related serine proteases and an inhibition constant (K_i_) of 0.4 nmol/l [10]. Importantly, rivaroxaban was shown to inhibit free, prothrombinase-associated and clot-associated Factor Xa without having a direct effect on platelet aggregation [15]. This is in contrast to the indirect Factor Xa inhibitors, such as fondaparinux, which do not inhibit Factor Xa that is bound to the prothrombinase complex [16]. Unlike rivaroxaban, fondaparinux also requires an antithrombin cofactor [9]. Because rivaroxaban does not inhibit thrombin directly, rivaroxaban does not affect the haemostatic function of pre-existing thrombin molecules [16].

Given the central role of thrombin in coagulation, direct targeting of this factor results in an antithrombotic effect. Thrombin is produced in small amounts in the initiation phase of the coagulation cascade but is generated in much greater amounts in the propagation phase and is essential to the amplification of coagulation and the formation of fibrin [14].

## Pharmacokinetic and pharmacodynamic properties of rivaroxaban and other novel oral anticoagulants in healthy volunteers and specific patient populations

### Phase I studies in healthy volunteers and special populations

In phase I studies in healthy subjects, rivaroxaban was found to have predictable PK properties (Table 1), with high absolute bioavailability after oral dosing [10,17], high and reversible plasma protein binding and a mean terminal half-life of 5–13 hours after a 10 mg dose [3,18]. Systemic clearance was low (approximately 10 l/h) [3]. Unchanged rivaroxaban was the predominant compound found in human plasma, and no major or active circulating metabolites were detected [19]. After single doses of 1.25–80 mg, which were well tolerated and did not lead to an increased risk of bleeding, peak plasma concentrations occurred within 2–4 hours of dosing, and maximal Factor Xa inhibition was seen after 1–4 hours [17]. This fast onset of action is similar to that of the low molecular weight heparins; for enoxaparin, maximum plasma anti-Factor Xa activity occurs 1–4 hours after injection [20]. The half-life of the biological effect of rivaroxaban was 6–7 hours, with Factor Xa inhibition still evident 24 hours after the administration of doses >5 mg [17]. Rivaroxaban was selective for Factor Xa inhibition and had no direct effect on thrombin and no effect on antithrombin activity [17]. When multiple doses up to 30 mg bid were given for 7 days, Factor Xa activity was inhibited in a dose-dependent manner, reaching a maximum after approximately 3 hours and continuing for at least 12 hours [21]. Peak plasma concentrations were reached after 3–4 hours and the terminal half-life was 5.7–9.2 hours at steady state, without accumulation at any dose [21]. The systemic clearance and volume of distribution in healthy volunteers are approximately 10 l/h and 50 litres, respectively, with moderate interindividual variability [3]. For single and multiple doses, prolongation of coagulation tests followed a similar profile to Factor Xa inhibition and correlated closely with plasma concentrations [22].

**Table 1:** Pharmacokinetic parameters (median values) of rivaroxaban, apixaban and dabigatran in healthy adults

**Agent**	**F**_**abs**_**, %**	**C**_**max**_**, ng/ml**	**AUC, ng · h/ml**	**t**_**max**_**, h**	**t**_**½**_**, h**	**V**_**d**_**, l**	**CL/F, l/h**	**Plasma protein binding, %**	**Renal excretion of unchanged drug, %**
Rivaroxaban	80–100 [3]	141* [17]	1020* [17]	2–4 [3]	5–13 [3]	50 [3]	10 [3]	92–95 [3]	33 [3]
Apixaban	50 [5]	460^†^[23]	4100^†^[23]	3–4 [5]	12 [5]	21 [5]	3 [5]	87 [5]	27 [5]
Dabigatran	6.5 [6]	110^‡^[24]	900^‡^[24]	1–2 [6]	12–17 [6]	60–70 [6]	105–170 [24]	34–35 [6]	85 [25]

*With 10 mg oral dose.

^†^With 20 mg oral dose.

^‡^With 150 mg oral dose.

Values rounded to nearest integer.

*AUC* area under the concentration–time curve, *CL/F* apparent clearance, *C*_*max*_ maximum plasma concentration, *F*_*abs*_ absolute bioavailability, *t*_*max*_ time to maximum concentration, *t*_*½*_ apparent half-life, *V*_*d*_ volume of distribution at steady state.

Several phase I studies were also conducted in special patient populations. These indicated that relevant PK and PD parameters remained consistent regardless of body weight [26], age [27,28], gender [28,29] or ethnicity [29,30], suggesting that dose adjustment is unnecessary. However, the presence of hepatic and renal impairment may lead to relevant PK/PD effects. For a 10 mg dose, mild hepatic impairment (Child–Pugh A) led to minimal PK and no PD changes compared with healthy controls, although moderate impairment (Child–Pugh B) led to significant increases in exposure and associated enhanced Factor Xa inhibition [3,31]; there are no data in patients with severe hepatic impairment. Approximately two-thirds of a rivaroxaban dose undergoes metabolic degradation, of which half is eliminated renally and half via the hepatobiliary route; the other one-third of the administered dose is excreted directly via the kidneys as unchanged, active drug, principally through active renal secretion via the transporter proteins P-glycoprotein (P-gp) and breast cancer resistance protein [3,19]. This gives an overall renal clearance of 3–4 l/h, meaning that decreasing renal function leads to an increase in rivaroxaban plasma concentrations and, correspondingly, PD effects [32]. No data exist for patients with creatinine clearance (CrCl) <min/min. As a result of high plasma protein binding (92–95% [3,18]), rivaroxaban is not expected to be dialysable [3].

Taking rivaroxaban with food did not have a significant effect on PK parameters for a 10 mg dose compared with fasting [3], but decreases in maximum plasma concentration (C_max_) and overall exposure (area under the concentration–time curve, AUC) were evident at a dose of 20 mg in the fasted state [33], as a result of decreased bioavailability and absorption rate with increasing dose [22]. When a dose of 20 mg was administered with food, complete bioavailability of rivaroxaban was restored [34]. Absorption was not affected by changes in gastric pH induced by ranitidine or antacid [33]. Because rivaroxaban is metabolised via cytochrome P450 (CYP) 3A4, CYP2J2 and CYP-independent mechanisms, and active renal secretion is mediated by P-gp and breast cancer resistance protein, co-administration with strong inhibitors of both CYP3A4 and P-gp, such as the azole antimycotic ketoconazole or the HIV protease inhibitor ritonavir, led to increased exposure and PD effects [3,35]. Strong inhibitors of one or the other, or moderate inhibitors of both of these pathways produced less marked effects [3,35]. Concomitant administration of rivaroxaban and strong CYP3A4 and P-gp inducers, such as the antibiotic rifampicin, led to decreases in PK and PD effects, whereas interactions with substrates of CYP3A4 and/or P-gp were considered not to be clinically relevant. Rivaroxaban does not inhibit or induce any major CYP isoforms, such as CYP3A4 [3]. Co-administration with enoxaparin produced an additive PD effect but did not affect the PK of rivaroxaban [3,36]. Clopidogrel did not affect rivaroxaban PK but did lead to a relevant increase in bleeding time in approximately one-third of healthy subjects, although this was not correlated with changes in platelet aggregation [37]. Co-medication with naproxen or ASA did not lead to clinically relevant prolongation of bleeding time overall [38], but some individuals exhibited pronounced PD effects with rivaroxaban plus ASA [39].

Apixaban and dabigatran exhibit broadly similar PK profiles to rivaroxaban, but with some notable exceptions (Table 1). Unlike apixaban and rivaroxaban, dabigatran is administered as a prodrug (dabigatran etexilate). The latter is a substrate of P-gp and its low oral bioavailability is determined by intestinal P-gp transporters [40]. Once absorbed, the prodrug is rapidly converted to dabigatran by esterases [40]. The volume of distribution of apixaban [5] is lower than that of rivaroxaban [3] and dabigatran [6], which in turn exhibit higher clearance and lower protein binding. An important difference between the drugs is also the proportion of each that is excreted via the kidneys; renal elimination of apixaban (approximately 27%; the majority is excreted via the hepatobiliary route) [5] is slightly lower than that of rivaroxaban [3], but that of dabigatran is considerably higher (85%) [25]. Unlike rivaroxaban [3] and apixaban [5], neither dabigatran nor its prodrug are metabolised by CYP-dependent mechanisms [6]; however, owing to dabigatran etexilate being a P-gp substrate, the effect of P-gp inhibitors on the bioavailability of dabigatran is stronger than on rivaroxaban elimination [6].

### Venous thromboembolism prevention in the orthopaedic setting

All three approved novel OACs underwent initial clinical evaluation for the prevention of VTE after elective hip or knee replacement surgery. This population experiences high levels of post-operative VTE in the absence of adequate thromboprophylaxis [41]. The rivaroxaban phase III clinical programme comprised four studies, RECORD1 [42], RECORD2 [43], RECORD3 [44] and RECORD4 [45], in more than 12,500 patients undergoing elective total hip replacement (THR) or total knee replacement (TKR). All four trials showed a significant efficacy benefit with rivaroxaban over the comparator enoxaparin regimens without an increase in major bleeding, and this was confirmed by pooled analyses [46,47]. The preceding phase II clinical trials (ODIXa-HIP [48], ODIXa-HIP2 [49], ODIXa-KNEE [50] and ODIXaHIP-OD [51]) of rivaroxaban for VTE prevention in patients undergoing THR or TKR collected data that were used to construct population PK/PD models to characterise the PK and PD properties of rivaroxaban in orthopaedic surgery populations [11,52]. One model compared rivaroxaban od and bid doses in patients undergoing THR and investigated the influence of patient demographic characteristics on PK and PD parameters [11]. Only doses that had demonstrated a favourable profile compared with enoxaparin, i.e. total daily doses of 5–20 mg, were considered. The other model included both THR and TKR patients from the phase II programme [52]. In both models, PK data were fed into a non-linear mixed effects model (NONMEM), which allows population estimates to be derived for PK and PK/PD parameters and quantifies both the interindividual and interoccasion variability of these parameters, as well as residual (unexplained) variability. Importantly, inputs to the model could then be modified to simulate the effect of different dosing regimens and population demographic factors (such as age, renal function and body weight) and the effect of co-medications [11,52].

In the first model described above, a total of 5743 samples from 758 patients (362 patients from the bid study and 396 patients from the od study) were included. Patient demographics were similar between the rivaroxaban od and bid study populations [11]. Observed rivaroxaban plasma concentrations (mean, 5/95 percentiles) are shown in Table 2. An oral, one-compartment model with a first-order rate constant was found to accurately describe the PK of rivaroxaban. The results of the model confirmed that rivaroxaban exhibited a predictable, dose-proportional PK profile in THR patients, as it had in healthy volunteers, with similar values for PK parameters (Table 3). The maximum plasma concentration (C_max_) for a 10 mg od dose was similar to that in healthy volunteers (median 125 ng/ml vs 141 ng/ml). Steady-state trough concentrations (C_trough_) were a median of approximately 9 ng/ml; this value was in the order of magnitude required for *in vitro* inhibition of Factor Xa activity, which supported the use of the rivaroxaban 10 mg od dose [11]. PK parameters were affected by body weight, study day, age, renal function, serum albumin and haematocrit, but the average of these effects remained within the overall variability of the population. The residual variability of the model was moderate (52.6%). PK/PD analysis of both the rivaroxaban od and bid data indicated that prolongation of prothrombin time (PT), measured using the STA® Neoplastine® CI Plus assay (Diagnostica Stago, Parsippany, NJ, USA), correlated strongly with rivaroxaban plasma concentrations [11].

**Table 2:** Concentration–time profiles for rivaroxaban in different patient populations observed in clinical studies [Bayer HealthCare Pharmaceuticals and Janssen Research & Development, LLC; data on file]

**Indication and rivaroxaban dose**	**VTE prevention:**	**DVT treatment:**	**Prevention of stroke in patients with AF and CrCl ≥50 ml/min:**	**Prevention of stroke in patients with AF and CrCl <50 ml/min:**	**Prevention of CV events in patients with ACS:**
	**10 mg od**	**15 mg bid for 3 weeks followed by 20 mg od**	**20 mg od**	**15 mg od**	**2.5 mg bid**
**Time after dosing (hours)**	**Concentration, μg/l (5/95 percentile)**	**Concentration, μg/l (5/95 percentile)**	**Concentration, μg/l (5/95 percentile)**	**Concentration, μg/l (5/95 percentile)**	**Concentration, μg/l (5/95 percentile)**
1	111 (75.1–177)	235 (164–361)	216 (152–316)	189 (134–281)	41.3 (23.5–65.9)
2	122 (90.6–195)	270 (189–419)	250 (177–361)	219 (157–317)	44.1 (26.7–69.5)
3	114 (82.3–186)	259 (180–405)	246 (172–361)	216 (153–317)	42.0 (25.9–66.4)
4	102 (75.8–164)	237 (161–369)	232 (157–349)	205 (141–309)	38.7 (23.3–63.3)
5	90.7 (62.2–143)	213 (145–339)	215 (140–333)	191 (127–297)	35.2 (20.2–59.1)
6	80.2 (51.8–125)	191 (123–311)	198 (123–318)	177 (111–286)	31.7 (17.4–55.5)
9	55.2 (30.5–96.0)	137 (71.3–240)	155 (81.9–276)	141 (74.9–254)	22.8 (10.4–45.2)
12	37.8 (15.2–76.1)	97.8 (42.9–190)	121 (53.4–242)	112 (50.0–225)	16.2 (6.11–36.6)
18	17.9 (4.85–49.9)	50.0 (16.0–124)	73.5 (22.0–187)	70.4 (21.9–180)	−
24	8.54 (1.36–37.2)	25.6 (5.93–86.9)	44.7 (9.02–147)	44.4 (9.42–143)	−

Plasma concentrations given as geometric mean values with 90% prediction intervals (5/95 percentiles). Values given to 3 significant figures.

*ACS* acute coronary syndrome, *AF* atrial fibrillation, *bid* twice daily, *CrCl* creatinine clearance, *CV* cardiovascular, *DVT* deep vein thrombosis, *od* once daily, *VTE* venous thromboembolism.

**Table 3:** Comparison of selected pharmacokinetic parameters (median values) with rivaroxaban in specific patient populations

**Population and rivaroxaban dose**	**C**_**max**_**, ng/ml**	**C**_**trough**_**, ng/ml**	**AUC, ng · h/ml**	**V**_**d**_**/F, l**	**Interindividual variability in V**_**d**_**, %CV**	**CL/F, l/h**	**Interindividual variability in CL/F, %CV**
Hip surgery patients, 2.5–10 mg bid; 10 mg od	125* [11]	9* [11]	1170^†^[11]	58 [11]	32 [11]	5–8 [11]	38 [11]
Patients treated for DVT; 10 mg bid, 20 and 30 mg od or bid, or 40 mg od	270 [12]	25^‡^[12]	2870 [12]	54 [12]	29 [12]	6 [12]	40 [12]
Patients with AF; 15 and 20 mg od	257^‡^ [Girgis. Unpublished data]	32 [12]	3466^†^ [Girgis. Unpublished data]	80 [Girgis. Unpublished data]	18 [Girgis. Unpublished data]	6 [Girgis. Unpublished data]	35 [Girgis. Unpublished data]
Patients with ACS; 2.5 mg bid	44^§^[86]	16^§^[86]	361^§^[86]	58 [86]	10 [86]	6.5 [86]	31 [86]

*10 mg od.

^†^AUC over a 24-hour period at steady-state.

^‡^20 mg od at steady-state.

^§^2.5 mg bid at steady-state; AUC over a 12-hour period.

Values rounded to nearest integer.

*AF* atrial fibrillation, *AUC* area under the concentration–time curve, *bid* twice daily, *C*_*max*_ maximum plasma concentration, *C*_*trough*_ steady-state trough concentration, *CL/F* apparent clearance, *CV* coefficient of variance, *DVT* deep vein thrombosis, *NR* not reported, *od* once daily, *V*_*d*_*/F* volume of distribution at steady state.

**Figure 2:**
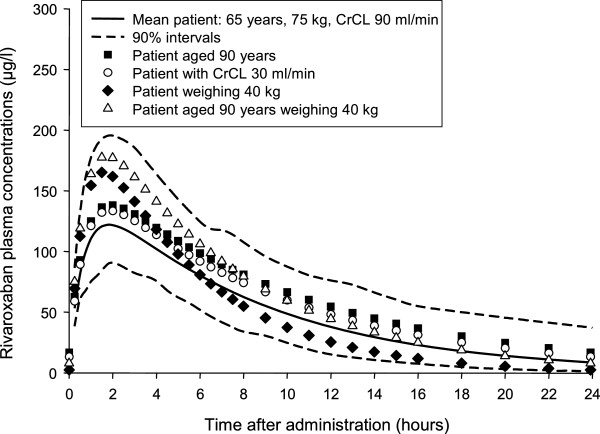
**Simulations of rivaroxaban plasma concentration–time profiles in typical patients compared with overall population estimates.** Typical patients are elderly (90 years), have moderate to severe renal impairment; CrCl 30 ml/min), have low body weight (40 kg), or are elderly with low body weight. Patients receiving rivaroxaban 10 mg once daily (mean with 90% interval) [11]. CrCL/CrCl, creatinine clearance. Reproduced with permission from Mueck W, Borris LC, Dahl OE *et al*. Population pharmacokinetics and pharmacodynamics of once- and twice-daily rivaroxaban for the prevention of venous thromboembolism in patients undergoing total hip replacement. *Thromb Haemost* 2008;100:453–461.

In order to provide insight into the expected influence of patient demographics, the model was then used to simulate the PK of rivaroxaban 10 mg od in patients with extreme characteristics; this included age 90 years, moderate to severe renal impairment (creatinine clearance [CrCl] 30 ml/min), low body weight (40 kg) and combined age 90 years and low body weight. The predicted plasma concentration–time profiles of rivaroxaban for the typical individual in each of the four demographic groups fell within the predicted 90% confidence intervals for the average population in these studies (Figure 2), confirming that rivaroxaban 10 mg od could be given without the need for dose adjustment in THR patients regardless of factors such as old age and moderate renal impairment [11]. In the second model, the only major difference between PK properties for TKR and THR was that clearance was 26% lower in the knee study, which led to ~30% greater exposure. The models exhibited moderate residual variability (37% and 34% in the hip and knee studies, respectively) [52].

In its phase III clinical programme, dabigatran 150 mg and/or 220 mg od were compared with enoxaparin regimens for the prevention of total VTE in patients who had undergone TKR (RE-MODEL [53] and RE-MOBILIZE [54] trials) or THR (RE-NOVATE [55] and RE­NOVATE II [56] studies). As with rivaroxaban, the doses used in these phase III trials were determined by a series of phase II studies, and one of these (BISTRO I) also provided PK data that could be used to construct a population PK model [57]. In total, 289 patients received dabigatran at doses of 150 or 300 mg od or 12.5–300 mg bid, and 4604 blood samples were available for use in the model [57]. Unlike for rivaroxaban, a two-compartment model most accurately described the PK profile of dabigatran, which demonstrated dose-proportionality and linear kinetics. The rate of drug absorption and apparent clearance during days 0 and 1 of treatment were significantly lower (p < 0.001) than on days 2–10, which may be explained by changes in gastrointestinal motility caused by surgical effects or co-medication; a similar finding was reported in the rivaroxaban models for this patient population. Weight, gender, variations in most laboratory measurements, smoking and alcohol consumption did not affect the PK of dabigatran but, unsurprisingly for a drug with 80% renal clearance, decreasing CrCl significantly increased plasma exposure [57]. Despite this, simulations indicated an apparent degree of overlap in concentration–time profiles for renally impaired patients compared with unimpaired controls, supporting the use of fixed dabigatran doses in patients undergoing THR regardless of demographic factors [57].

The phase III programme investigating apixaban for VTE prophylaxis after THR or TKR surgery consisted of three studies, ADVANCE-1, ADVANCE-2 and ADVANCE-3 [58-60], which compared apixaban 2.5 mg bid with standard enoxaparin regimens. No population PK model for apixaban in the orthopaedic setting has yet been published.

### Treatment of acute deep vein thrombosis and prevention of recurrent venous thromboembolism

VTE is a major global healthcare problem that carries a substantial morbidity and mortality burden in the general population [61,62]. The phase III EINSTEIN DVT [63] and EINSTEIN PE [64] studies assessed a single-drug approach using rivaroxaban against the standard dual-drug approach of enoxaparin overlapping with a VKA in patients with confirmed acute symptomatic DVT or PE. These studies found that rivaroxaban was as effective as this standard regimen with similar or better safety outcomes. An extension study (EINSTEIN EXT) indicated that long-term rivaroxaban was also more effective than placebo in preventing recurrent VTE [63]. Given that high rates of VTE recurrence in the acute phase of treatment were seen in previous studies [65,66], a higher dose of rivaroxaban (15 mg bid) was employed in the first 3 weeks of treatment. The basis for the chosen regimen (15 mg bid for 3 weeks followed by 20 mg od) stemmed from the outcomes from two phase II studies (EINSTEIN and ODIXa-DVT) [67,68], which demonstrated a greater reduction in thrombosis burden with bid compared with od dosing in the acute phase of treatment. These studies also collected PK data to produce a population model to characterise the PK/PD of rivaroxaban od and bid doses in patients with acute DVT, including evaluation of the influence of demographic factors.

**Figure 3:**
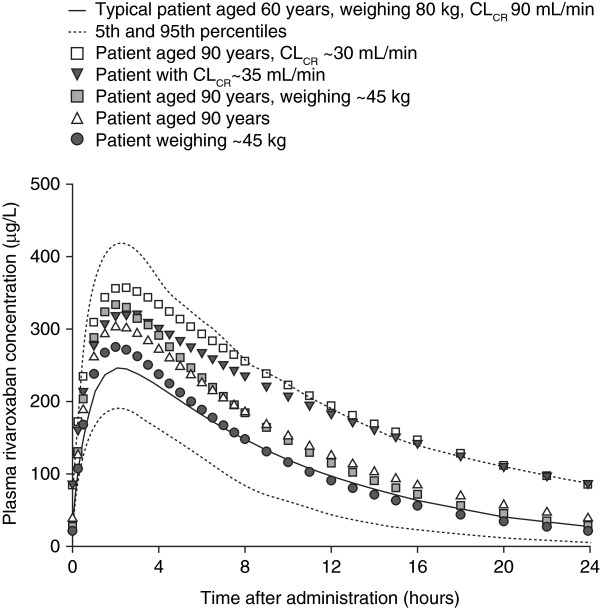
**Predicted plasma rivaroxaban concentration–time profiles for extremes in age, renal function and body weight.** Patients receiving rivaroxaban 20 mg once daily. The simulated patients had typical mean characteristics (age 60 years, body weight 80 kg, CrCl 90 ml/min) unless specified otherwise [12]. CL_CR_/CrCl, creatinine clearance. Reproduced from Mueck W, Lensing AW, Agnelli G *et al*. Rivaroxaban: population pharmacokinetic analyses in patients treated for acute deep-vein thrombosis and exposure simulations in patients with atrial fibrillation treated for stroke prevention. *Clin Pharmacokinet* 2011; 50:675–686 with permission from Adis (© Springer International Publishing AG 2011. All rights reserved).

**Figure 4:**
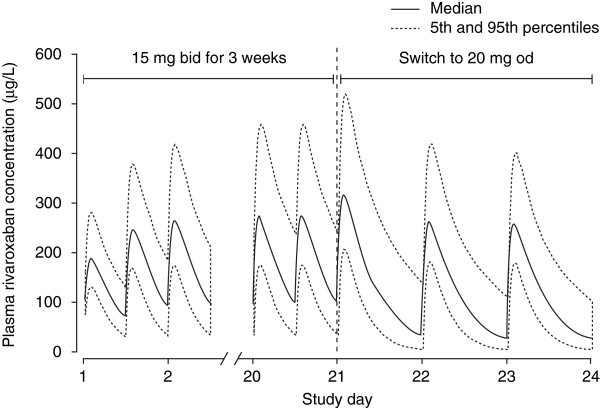
**Simulated venous thromboembolism treatment dosing regimen of rivaroxaban.** Regimen is 15 mg bid for 3 weeks followed by 20 mg od (n=870) [12]. bid, twice daily; od, once daily. Reproduced from Mueck W, Lensing AW, Agnelli G *et al*. Rivaroxaban: population pharmacokinetic analyses in patients treated for acute deep-vein thrombosis and exposure simulations in patients with atrial fibrillation treated for stroke prevention. *Clin Pharmacokinet* 2011; 50:675–686 with permission from Adis (© Springer International Publishing AG 2011. All rights reserved).

Data input into the model comprised 4634 rivaroxaban plasma samples from 870 patients [12]. Observed rivaroxaban plasma concentrations (mean, 5/95 percentiles) are shown in Table 2. As for healthy subjects and patients who had undergone major orthopaedic surgery, rivaroxaban PK was well described by a one-compartment model. Median C_max_, C_trough_ and AUC values at steady state at the daily dose chosen for the phase III programme (20 mg od) were correspondingly higher than for a 10 mg od dose (Table 3), and rivaroxaban exhibited the same previously documented dose-proportional PK profile. Age and renal function moderately affected the PK profile, but variations were within the overall variability seen in the studies (Figure 3) [12]. Variations in gender and body weight had a minimal effect. Simulations of the approved rivaroxaban dosing regimen for VTE treatment (15 mg bid for 3 weeks followed by 20 mg od) demonstrated that no fluctuations in C_max_ would be expected during the transition from bid to od dosing (Figure 4). Co-administration of laxatives, diuretics, non-steroidal anti-inflammatory drugs and ASA did not significantly alter the PK profile of rivaroxaban, although use of concomitant strong CYP3A4 inducers reduced rivaroxaban exposure by up to 50% [12]. As seen for patients undergoing orthopaedic surgery, an almost linear correlation between rivaroxaban exposure and PT prolongation was demonstrated [12].

Dabigatran has been evaluated for the treatment of acute VTE in the RE-COVER and RECOVER II clinical trials [69,70]. In these studies, patients received either parenteral anticoagulant followed by dabigatran 150 mg bid or parenteral anticoagulant overlapping with a VKA until an international normalised ratio (INR) of 2–3 was achieved. The dual-drug regimen with dabigatran was shown to be as effective as standard therapy, with a similar safety profile. Two further studies have shown dabigatran 150 mg bid to be effective for the long-term treatment of VTE, although the results of one study showed that dabigatran significantly increased the incidence of ACS compared with warfarin [71,72].

Apixaban is being evaluated for VTE treatment in two phase III clinical trials. The AMPLIFY study will compare apixaban 10 mg bid for 7 days followed by 5 mg bid with standard therapy for the treatment of acute VTE (http://www.clinicaltrials.gov; NCT00643201). AMPLIFY-EXT evaluated apixaban 2.5 mg bid or 5 mg bid versus placebo for 12 months after initial treatment of VTE. The recently published results demonstrated a significant reduction in recurrent VTE with apixaban without an increase in major bleeding [73].

### Stroke prevention in patients with non-valvular atrial fibrillation

Partially as a result of an ageing population, AF is now the most commonly occurring heart arrhythmia [74,75] and is a major risk factor for stroke [74]. The phase III ROCKET AF study showed that rivaroxaban (20 mg od) was non-inferior to dose-adjusted VKA therapy in patients with AF for the prevention of stroke and systemic embolism, with similar rates of bleeding [76]. In the secondary efficacy analysis in the as-treated safety population, rivaroxaban demonstrated superiority compared with warfarin. Importantly, rates of intracranial haemorrhage were significantly lower with rivaroxaban, and the incidences of myocardial infarction, vascular death or all-cause mortality were numerically lower [76]. Observed rivaroxaban plasma concentrations (mean, 5/95 percentiles) are shown in Table 2. A population PK model for rivaroxaban in patients with AF was constructed based on data from the DVT treatment studies, taking into account the fact that AF populations are typically older than those undergoing orthopaedic surgery or requiring treatment for VTE [12]. A virtual population of 1000 elderly patients with AF was simulated; results indicated that the average C_max_ and AUC of rivaroxaban in patients with AF could be expected to be slightly higher (7.4% for C_max_; 15.3% for AUC) than values for patients with DVT [12]. Renal impairment was expected to lead to increased exposure [12]. These predictions were subsequently confirmed in a population PK analysis of the ROCKET AF dataset, which included data from 161 individuals. Apparent clearance and volume of distribution at steady state were estimated to be approximately 6 l/h and 80 litres, respectively, with moderate interindividual variability (Table 3). PK parameters for patients with moderate renal impairment given rivaroxaban 15 mg od were generally similar to those estimated for patients with either no or mild renal impairment who received rivaroxaban 20 mg od. Ratios of the means of C_max_ and AUC for time 0 to 24 hours (AUC_0–24_) were 0.88 and 0.91, respectively, for patients with moderate renal impairment compared with those with mild impairment or normal renal function, and the distributions overlapped considerably, supporting the use of a reduced rivaroxaban 15 mg od dose for patients with moderate renal impairment [I Girgis. Unpublished data]. In addition, modelling work supported the use of a lower rivaroxaban dose in Japanese patients with AF [77,78], and in a phase III randomised study (J-ROCKET AF) a 15 mg od dose was found to be non-inferior to warfarin (hazard ratio 1.11; 95% confidence interval 0.87–1.42) for the prevention of stroke and systemic embolism [79].

Dabigatran was evaluated for the prevention of stroke in patients with AF in the phase III RELY study. In RE-LY, dabigatran 150 mg bid significantly reduced the risk of stroke or systemic embolism compared with warfarin, with a similar rate of major bleeding, whereas the 110 mg bid dabigatran dose had similar efficacy to warfarin and with significantly lower rates of major bleeding [80,81]. Data from phase I and II studies of dabigatran in healthy subjects (n = 128) and patients undergoing orthopaedic surgery or with AF (n = 2252) were used to construct a population PK model for dabigatran, which was then validated by comparison with PK data collected in RE­LY [82]. Given the high renal clearance of dabigatran, CrCl was built into the baseline assumptions for the model, and PD data were evaluated with the use of activated partial thromboplastin time (aPTT) assays. For the development of a PK model for dabigatran, a dataset was collated from 80 healthy volunteers (44% of whom had some level of renal impairment) and 1965 patients [82]. For both patients and healthy controls, the PK of dabigatran was best described by a two-compartment model and there was a linear correlation between clearance and renal function. Age, gender and therapeutic indication (AF or orthopaedic surgery) influenced clearance, and body weight influenced volume of distribution [82]. The values predicted by the model agreed well with those observed in RE-LY, and there was a linear correlation between dabigatran exposure and aPTT prolongation. Using the data to simulate plasma concentration–time profiles at steady state for a typical male patient with AF (age 68 years, weight 80 kg, CrCl 87 ml/min) controlling for a variety of covariates, the effect remained within the overall variability for the average patient with AF, with the exception of renal impairment, which led to increases in exposure of 40% for a CrCl of 50 ml/min and 90% with a CrCl of 30 ml/min. There was less peak-to-trough fluctuation with dabigatran dosing at 150 mg bid than for 300 mg od [82]. Further simulations of patients with moderate and severe renal impairment indicated that, compared with patients with moderate renal impairment (CrCl >30–49 ml/min) given dabigatran 150 mg bid, those with severe impairment (CrCl 15–30 ml/min) had a 35% higher average C_max_ with dabigatran 150 mg od and a 42% lower average C_trough_ with a dabigatran 75 mg od dose. Dabigatran 75 mg bid resulted in a reasonable matching of exposures [83].

### Secondary prevention of major cardiovascular events in patients with acute coronary syndrome

In the recent phase III ATLAS ACS 2 TIMI 51 trial, rivaroxaban 2.5 mg bid or 5 mg bid was shown to significantly reduce the incidence of death, myocardial infarction or stroke compared with placebo when added to standard dual anti-platelet therapy (ASA plus either clopidogrel or ticlopidine) in patients with ACS [84]. Using data from the preceding phase II study, ATLAS ACS TIMI 46 [85], a population PK model was constructed to characterise the parameters of rivaroxaban in this population and model the effects of demographic variations on rivaroxaban PK [86]. Data from 2290 patients were used and the observed rivaroxaban plasma concentrations (mean, 5/95 percentiles) are shown in Table 2. As for the models in other patient populations, rivaroxaban PK was described by an oral one-compartment model. Apparent clearance and volume of distribution were approximately 6.5 l/h and 58 litres, respectively, with low-to-moderate interindividual variability. Variations in renal function, age and body weight on exposure were consistent with previous findings and PK parameters were similar to those estimated for other patient populations (Table 3) [86].

The phase III APPRAISE-2 trial of apixaban demonstrated that in high-risk patients after ACS the addition of apixaban 5 mg bid to antiplatelet therapy increased rates of major bleeding without significantly reducing the rates of recurrent ischaemic events [87]. Dabigatran is not being investigated in phase III studies of patients with ACS.

## Laboratory monitoring of novel oral anticoagulants

Because novel OACs competitively and directly inhibit specific factors of the coagulation cascade, their concentration–time profiles directly determine the time course of inhibition (taking into consideration fluctuations with absorption and elimination processes associated with oral dosing). This is in contrast to warfarin, which works indirectly by inhibiting components necessary for the synthesis of blood coagulation factors; therefore, inhibition by warfarin is determined by the half-life of synthesis. The predictable PK and PD profiles of rivaroxaban and other novel OACs mean that routine coagulation monitoring is not normally required [88]. However, in cases where an emergency intervention requires immediate assessment of anticoagulation, such as prior to urgent surgery, it may be useful or essential to be able to measure the anticoagulant effect of a novel OAC. Available tests can be divided into assays that measure general clot formation and those that directly quantify inhibition of a specific clotting factor. Owing to their modes of action, novel OACs affect some of these assays in different ways and an understanding of this is key to correctly interpreting results.

### Clot-based assays

The most commonly available clot-based assays include the PT, dilute PT, aPTT, ecarin clotting time (ECT), HepTest and prothrombinase-induced clotting time (PiCT) [89]. Each of these tests measures the time taken for a plasma sample to form a clot after the addition of calcium and an activator in the presence of the anticoagulant to be monitored. This means that they are not specific to any particular anticoagulant. The effects of rivaroxaban, dabigatran and apixaban on these tests are summarised in Table 4.

**Table 4:** **Comparison and suitability of laboratory assays for monitoring novel oral anticoagulants [**[89]**,**[90]**]**

**Agent**	**PT**	**Dilute PT**	**aPTT**	**ECT**	**HepTest**	**PiCT**	**Chromogenic assays**	**Preferred assay**	**Assays that may be used in the absence of preferred assay**
Rivaroxaban	Dose-dependent prolongation but results vary with thromboplastin reagent	Results vary as for PT	Not as sensitive as PT	NA	Dose-dependent response with short incubation time	Sensitive if incubation avoided and human Factor Xa used	Dose-dependent response with human Factor Xa and buffer	Factor Xa chromogenic assay (with appropriate calibration)	PT (Neoplastin)
Apixaban	Prolongs PT but results may vary with thromboplastin reagent	More sensitive than PT	Other tests may be more sensitive	NA	Dose-dependent response and more sensitive than PT, dilute PT and aPTT	NR	Dose-dependent response	Rotachrom® Factor Xa chromogenic assay (with appropriate calibration)	Modified PT
Dabigatran	Prolongs PT but insensitive and results may vary with thromboplastin reagent	NR	More sensitive than PT but results vary with different reagents	Prolongs ECT in a dose-dependent manner	Prolongs HepTest but may be unsuitable	Prolongs PiCT but insensitive at lower doses	In development	Hemoclot suggested	ECT, aPTT

*aPTT* activated partial thromboplastin time, *ECT* ecarin clotting time, *NA* not applicable, *NR* not reported, *PiCT* prothrombinase-induced clotting time, *PT* prothrombin time.

PT measures the time for plasma to clot after the addition of calcium and thromboplastin. The results are always given in seconds. Rivaroxaban prolongs PT in a dose-dependent manner but the extent of prolongation depends on the thromboplastin reagent used [90,91]. A multicentre study that evaluated the interlaboratory variability of PT measurements with rivaroxaban found that the use of local reagents led to greater variability than when all laboratories used a standardised Neoplastine CI Plus test (Diagnostica Stago, Asnières-sur-Seine, France), which had a higher sensitivity for rivaroxaban than many other PT assays [92]. Another group has also recently reported reproducible results with a calcium chloride-modified PT assay [93]. The variation in the sensitivity of different thromboplastin reagents is overcome for VKA monitoring by conversion to the INR, but this is specific to VKAs and cannot be used with rivaroxaban or the other novel OACs [91].

Even with standardisation, there are a number of other limitations of PT when applied to novel OACs [89,90]. The presence of concomitant systemic conditions, such as hepatic impairment, sepsis or vitamin K deficiency, can lead to a prolongation of PT. The PT is dependent on factors of the extrinsic coagulation pathway other than Factor Xa and it is not specific to any agent. Furthermore, the short half-life of, for example, rivaroxaban (5–13 hours) led to transient PT results, whereas for warfarin (half-life 36–42 hours) less variability can be expected. In addition, disappearance from the plasma of a Factor Xa inhibiting agent does not always correlate with a return to normal Factor Xa levels. Finally, PT reagents are insensitive at low concentrations of rivaroxaban [89,90] and are not able to accurately measure the C_trough_ levels predicted for rivaroxaban in PK models: C_trough_ levels were in the range 9–32 ng/ml (Table 3) but Neoplastine Plus can only measure plasma levels down to approximately 50 ng/ml, meaning that a PT reading taken around the time of C_trough_ will likely provide a false negative result [89]. These limitations also apply to dilute PT assays. Nevertheless, if used in an emergency and in the absence of any other available test, Neoplastine Plus (with results expressed in seconds) is the recommended agent for assessing the anticoagulant effect of rivaroxaban [3].

The aPTT test is performed in the absence of a tissue factor and, therefore, measures the overall function of the intrinsic coagulation pathway. Traditionally performed to monitor coagulation with unfractionated heparin, aPTT is performed by adding a contact activator (e.g. celite, ellagic acid, kaolin or silica) and cephalins to citrated plasma [89]. Calcium is added after a preincubation period and the clotting time is then measured. When using aPTT to assess rivaroxaban and apixaban, the test was less sensitive than PT, and results with dabigatran were non-linear at higher doses [89,90]. A recent study that tested a range of clotting assays with dabigatran suggested that aPTT could be used as a screening test for the risk of overdose but not for quantitative measurement of dabigatran [94].

HepTest is a clot-based anti-Factor Xa assay, in which a plasma sample is preincubated with bovine Factor Xa before addition of calcium chloride and thromboplastin. Rivaroxaban prolongs HepTest clotting time, although a shortened incubation time must be used with low concentrations to ensure a linear dose–response [91]. In contrast, ECT measures thrombin clotting using a derivative of snake venom to generate a prothrombin intermediate, and is thus likely to be more appropriate for monitoring dabigatran activity. Indeed, dabigatran prolongs ECT in a dose-dependent manner and can be calibrated to dabigatran concentrations [95]. The thrombin generation test is capable of assessing each phase of thrombin generation but may lack sensitivity [89], whereas another assay, Hemoclot, has been reported to have high sensitivity, good reproducibility and a linear dose correlation with dabigatran [90,94]. The PiCT test, which uses Factor Xa, phospholipids and a Factor V activator, is affected by both Factor Xa and thrombin inhibitors. Rivaroxaban and dabigatran prolong PiCT, although incubation should be avoided with rivaroxaban and human, rather than bovine, Factor Xa must be used [89]. However, as with many of the other tests described, a lack of sensitivity at low concentrations and variation between tests limit their usefulness for assessing novel OACs [89].

### Chromogenic assays

Chromogenic assays measure the change in absorbance when a chromophore-tagged substrate of a specific clotting factor is cleaved by the coagulation factor to be measured, a process that is inhibited by the presence of the anticoagulant [89]. Such assays are more specific than clot-based assays and have proved suitable for quantitative measurement of rivaroxaban exposure with dose-dependent results covering both the expected C_max_ and C_trough_ levels after therapeutic doses. Nevertheless, appropriate calibration over a wide range of plasma concentrations is required to create a standard reference dose–response curve [89,90]. A recent *ex vivo* study evaluated the accuracy of three anti-Factor Xa chromogenic assays for measuring rivaroxaban concentrations using plasma samples from healthy subjects and patients [96]. Assays that did and did not include exogenous antithrombin, with two different concentration calibration sets for each, were investigated. All assays showed a linear relationship between actual rivaroxaban concentrations and the optical density of the chromogenic assays. However, although the non-antithrombin assays were able to provide accurate results over a wide range of rivaroxaban concentrations, the assay that contained exogenous antithrombin provided falsely elevated results, suggesting that it is unsuitable for use with rivaroxaban [96].

A recent field trial investigated the inter-laboratory viability of the measurement of rivaroxaban plasma concentrations with anti-Factor Xa chromogenic assays. The study was conducted in 24 laboratories in Europe and North America using standardised rivaroxaban calibrators and plasma control samples [97]. Each centre used both the centrally provided modified STA® Rotachrom® assay (Diagnostica Stago) and local Factor Xa reagents to perform tests on a variety of sample concentrations. Using the centrally provided assay, a lower inter-laboratory variation was found compared with when local reagents were employed, with the greatest difference found at lower concentrations of rivaroxaban. This study suggests that, using standard calibrators and controls, a range of rivaroxaban plasma concentrations (20–660 ng/ml), which covers the expected rivaroxaban plasma levels after therapeutic doses, could be measured using the chromogenic anti-Factor Xa assay STA Rotachrom [97]. This assay could provide a more sensitive and specific alternative to the previously described PT method for the measurement of rivaroxaban plasma concentrations. The STA Rotachrom assay and two other chromogenic assays, Biophen DiXaI® (Hyphen Biomed) and Technochrom® anti-Xa (Technoclone), have received European authorisation for commercial distribution. STA Rotachrom has been identified as the preferred assay for measuring the activity of apixaban [5]. Chromogenic assays to measure dabigatran are currently in development.

## Discussion

Of the three currently licensed novel OACs, the PK and PD profile of rivaroxaban is arguably the most fully elucidated. Observations in phase I and phase II studies have shown that rivaroxaban has predictable, dose-proportional PK with an anticoagulant effect that also increases in a linear fashion with increasing plasma concentration; importantly, this profile is largely consistent in the presence or absence of demographic variations [22]. Although characterisation of PK in healthy subjects is important, it is vital to understand how a drug is likely to behave in actual patient populations. The use of population PK modelling is an invaluable tool for achieving full PK and PD characterisation. There is now a substantial body of patient modelling data published for rivaroxaban and dabigatran, although this is currently lacking for apixaban. One advantage of PK modelling is that it can be used to simulate ‘extreme’ scenarios, such as those that present in patients who are elderly, have renal or hepatic impairment, or who are obese. The models described herein show that, for the most part, the PK and PD profiles of rivaroxaban and dabigatran remain within acceptable boundaries for most individual patients, supporting the use of fixed dosing regimens [11,12,52,57,82]. Overall, both agents exhibit only moderate PK/PD variability, which contrasts with the unpredictable interindividual variations seen with warfarin that necessitate routine coagulation monitoring.

The other novel OACs also exhibit similarly predictable profiles, but some comparative PK properties differ in ways that may be important to consider in a given clinical situation. For example, the high renal clearance of dabigatran (80%) means that it is not considered suitable in the EU for patients with severe renal insufficiency (CrCl 15– < 30 ml/min) [6], although it can be used for the prevention of stroke in severely renally impaired patients with AF in the US (at a reduced 75 mg od dose) [8]. Rivaroxaban and apixaban, which are eliminated in greater proportions via other non-renal routes, may be used with caution in the relevant licensed indication in such patients [3,5]. Equally, the drug interaction profile of each agent may be an important consideration if a patient is taking concomitant medications. Rivaroxaban should not be used in conjunction with strong inhibitors of both CYP3A4 and P­gp, such as azole-antimycotics (e.g. ketoconazole, itraconazole, voriconazole and posaconazole) or HIV protease inhibitors (e.g. ritonavir) because competitive elimination with rivaroxaban will increase exposure to the latter to a clinically relevant degree [3,35]. However, comedication with strong inhibitors of one of these pathways only, or moderate inhibitors of both, may be considered with caution. In contrast, dabigatran is not metabolised via CYP3A4 pathways but should not be co-administered with potent P-gp inhibitors (e.g. amiodarone) [6], and apixaban should not be given in conjunction with strong CYP3A4 inhibitors but is not affected by competition for P-gp transport [5].

An important observation derived from population PK modelling is the effect of renal clearance and age on the PK/PD profiles of rivaroxaban and dabigatran. These two parameters are often linked because renal efficiency decreases with advancing age. Results of PK modelling with dabigatran led to recommendations for a reduced dose of 150 mg od to be used for VTE prevention in patients with moderate renal impairment (CrCl 30–49 ml/min) [6,83]. Rivaroxaban modelling confirmed that no dose reduction is needed for patients with CrCl 15–49 ml/min (moderate or severe renal impairment) receiving the 10 mg dose approved for VTE prevention after elective hip and knee replacement surgery. For the treatment of DVT in patients with CrCl 15–49 ml/min, rivaroxaban PK modelling supported the recommended 15 mg bid dose for the acute phase (first 21 days) of therapy and a reduced dose of rivaroxaban 15 mg od for the extended treatment phase (post-day 21). For the prevention of stroke and systemic embolism in patients with non-valvular AF and CrCl <50 ml/min, the reduced dose of rivaroxaban 15 mg od was supported by phase III trial data (CrCl 30–49 ml/min; moderate renal impairment) and modelling (CrCl 15– < 30 ml/min; severe renal impairment) [3,76]. Given that data are limited, rivaroxaban should be used with caution in patients with CrCl 15– < 30 ml/min in all settings [3].

Although not routinely required, laboratory testing of anticoagulant activity may sometimes be necessary when employing novel OACs. Because of the PK/PD properties of these agents, few clot-based assays are appropriate for this task, and in general there is a lack of standardised calibration and methodology for conducting these tests with the novel OACs [90,95]. This contrasts to the case with warfarin, for which cumulative years of experience have led to a standardised calibration of PT test results using the INR. At clinically relevant plasma concentrations of rivaroxaban, the effect on PT prolongation is small and short-lived, and the test has a low sensitivity for all novel OACs [90,95]. However, the advantage of PT is that it is a standard test that is available and can be performed rapidly in most clinical laboratories worldwide [92]. Chromogenic assays that respond to specific coagulation factors are now available and can provide specific, sensitive and accurate quantitative measurement of rivaroxaban exposure within the expected range of plasma concentration seen with therapeutic doses [90,95].

For this reason, approved chromogenic assays should be regarded as the gold standard for measuring rivaroxaban plasma levels under the unusual circumstances where this may be required. It is important to note that, regardless of the assay used, the result will provide an indication of drug plasma levels but not a direct measurement of anticoagulation. Therefore, chromogenic assays should only be used to establish the presence or absence of the drug in plasma; the potential influence of the time of drug administration should be taken into consideration when interpreting the results. This differs from INR monitoring for VKAs where a safe and effective range is targeted and the INR results ultimately drive treatment decisions. With the anticipated increase in the use of novel OACs, laboratories should familiarise themselves with chromogenic assays and how the results should be interpreted.

## Conclusions

Rivaroxaban and dabigatran (and apixaban based on such data as have been published) have predictable PK and PD properties allowing for fixed oral dosing regimens to be followed regardless of demographic variations without the need for routine coagulation monitoring in most cases. The relatively short half-life of these drugs and the direct correlation between concentration–time profile and inhibition are advantages for fast offset of action when treatment is stopped. In circumstances where the measurement of plasma concentration is required, the use of standardised calibrators and controls is essential to obtaining accurate data – this appears possible with anti-Factor Xa chromogenic assays for rivaroxaban and apixaban, and HemoClot, ECT or aPTT for dabigatran. However, it is important that the results of any of these assays are interpreted within the context of the time of drug administration.

## Abbreviations

ACS: Acute coronary syndrome; AF: Atrial fibrillation; aPTT: Activated partial thromboplastin time; ASA: Acetylsalicylic acid; AUC: Area under the concentration–time curve; bid: Twice daily; CrCl: Creatinine clearance; CYP: Cytochrome P450; DVT: Deep vein thrombosis; ECT: Ecarin clotting time; INR: International normalised ratio; OAC: Oral anticoagulant; od: Once daily; P-gp: P-glycoprotein; PD: Pharmacodynamic; PiCT: Prothrombinase-induced clotting time; PK: Pharmacokinetic; PE: Pulmonary embolism; PT: Prothrombin time; THR: Total hip replacement; TKR: Total knee replacement; VKA: Vitamin K antagonist; VTE: Venous thromboembolism.

## Competing interests

The authors are employees of Bayer HealthCare Pharmaceuticals, the manufacturers of rivaroxaban.

## Authors’ contributions

All authors contributed to the drafting and review of the manuscript, and read and approved the final manuscript.
